# Techno-Functional and Rheological Properties of Alternative Plant-Based Flours

**DOI:** 10.3390/foods12071411

**Published:** 2023-03-26

**Authors:** Celia Badia-Olmos, Laura Laguna, Claudia Mónika Haros, Amparo Tárrega

**Affiliations:** Instituto de Agroquímica y Tecnología de los Alimentos (IATA-CSIC), Agustín Escardino Benlloch 7, 46980 Valencia, Spainlaura.laguna@iata.csic.es (L.L.);

**Keywords:** plant-based flour, protein, techno-functional properties, rheology

## Abstract

The use of alternative vegetal sources is a proposed strategy to improve the diversity and quality of plant-based products on the market, currently led by soy and pea. This study compares the techno-functional properties of seven vegetable flours (chickpea, lentil, red lentil, white bean, quinoa, amaranth, and oat) and the rheological properties of their flour pastes and gels. All techno-functional properties significantly (α = 0.05) varied depending on the type of flour. Among the flours studied, the highest swelling capacity was for white bean and the lowest for chickpea and red lentil. Water holding capacity was high for white bean and oat flours and low for red lentil. Oat and quinoa flours had the highest oil-holding capacity. Emulsifying and foaming capacities were high for all pulse flours but poor for amaranth and oat flours. However, amaranth and oat provided a much higher viscosity during heating than the rest of the flours. The viscoelastic properties of the flour pastes indicated that they all had a gel structure with storage modulus (G′) values over loss modulus (G″) values. From the viscoelastic properties, amaranth and quinoa showed a weak gel structure with low G′ and G″ values, and the chickpea, lentil, and red lentil formed pastes with a high elastic contribution (high G′ values). In agreement, these three pulse flours were the only ones able to form hard, self-standing gels. These results show the potential of vegetal flours from alternative sources in the development of new plant-based products.

## 1. Introduction

There is increasing interest in plant-based food products as consumers become more aware of the need to change to more sustainable and healthy food consumption [1]. Reducing animal-based consumption in our diet requires alternative products that provide enough protein to maintain an adequate intake. Therefore, vegetable proteins have received a lot of attention as ingredients in the formulation of new plant-based products.

Proteins are interesting not only because of their nutritional value but also because of their techno-functional properties, which usually play a relevant role in the elaboration process and are responsible for the final structure of the product. For years, both research and industry have focused mainly on two high-protein vegetable sources: soy and pea [2,3]. Research has shown that these vegetable proteins have good water retention, emulsifying, foaming, and gelling activities, which can also be modulated by factors such as pH and ionic strength [4,5]. This has allowed the development of most plant-based alternatives, although the low variety and sensory quality of plant-based alternatives remain some of the main barriers for consumers. However, there are other more sustainable and protein-rich sources that could provide an opportunity to develop new plant-based products. For example, pulses (chickpea, lentil, red lentil, and white bean) are produced worldwide and are a good source of sustainable protein that is underutilised [2]. Additionally, there are cereals such as oats with a high protein content [6] or pseudocereals such as amaranth and quinoa that are less consumed but have potential nutritional benefits due to their well-balanced content in amino acids [7]. Protein isolates and concentrates, which have been extensively studied, are used as ingredients to enrich or elaborate products. However, conventional methods of protein extraction are expensive, long, or include chemical solvents that may affect protein functionality [8]. Using flour, obtained using a mild method such as milling, results in a less expensive, more sustainable, and natural ingredient that can better favour the development of new products. Furthermore, consumers’ increasing interest in less processed products and ingredients would be achieved this way.

In addition, to protein, flour also includes carbohydrates (starch and fibre) and lipids that may also influence its techno-functional properties [9]. Starch can gelatinise under heating and has an important structural role in determining the rheological properties of a final product. The techno-functional properties provided by these two major components and their interactions will determine their suitability as the base ingredient of new products or concepts.

Recent papers have studied the different properties of pulse flours [10,11], amaranth flour [12], gels of quinoa flour [13], and oat flour [14]. However, no previous studies have compared the same properties of flours from these different and lesser-used sources in a single study, also considering the rheological properties of products formed after heating.

Therefore, the objective of this research is to determine and compare the techno-functional properties of seven plant-based flours (chickpea, lentil, red lentil, white bean, quinoa, amaranth, and oat) and the rheological properties of their pastes and gels.

## 2. Materials and Methods

### 2.1. Materials

Chickpea, lentil, red lentil, white bean, and quinoa flours were supplied by the Dacsa Group (Valencia, Spain); amaranth and oat flours were purchased from Tentorium Energy S.L. (Tarragona, Spain). The flours were all wholegrain, except for red lentils, which came from dehulled seeds. The granulation (particle size distribution) and the moisture content of each flour are presented in Table 1.

### 2.2. Proximate Flour’s Composition

Total dietary fibre content was determined according to the AOAC method 991.43 [15] with a total dietary fibre assay kit (K-TDFR-200A, Megazyme International Ltd., Wicklow, Ireland). Briefly, the flour sample (1 g) was suspended in a MES/TRIS buffer. Subsequently, thermostable α-amylase (50 µL), protease (100 µL), and amyloglucosidase (200 µL) were added to digest the sample and remove starch and protein. The fibre of the sample was precipitated and filtered with ethanol and finally dried overnight at 103 °C. Total dietary fibre content was obtained and corrected for residual protein content and ash.

The fat content was determined according to the AACC method 30.10 [16]. The extraction of the sample was performed with petroleum ether using the Soxhlet technique. The ether was evaporated, and the lipid residue was weighed and expressed as a percentage of the fat content.

Protein content determination was performed using the Dumas combustion procedure according to ISO (International Organization for Standardization)/TS (Technical Specification) 16634-2 [17]. The total nitrogen content was measured using a thermal conductivity cell in a Rapid N exceed analyser (Elementar, Langenselbold, Germany). The percentage of nitrogen was obtained and converted to protein content using the conversion factor of 6.25.

The carbohydrate content was calculated by subtracting the moisture (%), fat (%), protein (%), and total dietary fibre (%) content from 100.

All measurements were performed in duplicate.

### 2.3. Swelling Capacity of Flours

Swelling capacity of flours determination was based on the method proposed by Robert son et al. (2000) [18]. The flour (1 g) was manually mixed with 10 mL of water in a 10 mL graduated cylinder and allowed to rest for 20 h at room temperature (23 °C). The bed volume (mL) was recorded, and the swelling capacity of the flour was expressed as the volume occupied by the hydrated flour. For each flour, the measurements of swelling capacity were conducted in triplicate.

### 2.4. Water Holding Capacity and Oil Holding Capacity of Flours

For determining the water holding capacity and oil holding capacity, the method proposed by Azia and Komathi (2009) [19] was followed. Three grammes of flour were mixed with 30 mL of water for water holding capacity and with 30 mL of sunflower oil for oil holding capacity. The solutions were vortexed for 30 s and allowed to stand for 2 h for water holding capacity and 1 h for oil holding capacity at room temperature (23 °C). Samples were centrifuged at 388× *g* for 10 min (Sorvall RC-5B centrifuge, DuPont Company, Newtown, CT, United States), and the supernatant was carefully removed and the resulting pellet was weighed. The results of water and oil holding capacity were calculated by deducting the weight of the dry sample from the weight of the resulting pellet and dividing the result by the weight of the dry sample. The final results were expressed as the g of water (water holding capacity) or g of oil (oil holding capacity) retained per g of flour. For each flour, measurements of water holding capacity and oil holding capacity were conducted in triplicate.

### 2.5. Emulsifying Capacity of Flours

The emulsifying capacity of flour was determined based on the methodology described by Yasumatsu et al., 2014 [20]. Flour (1 g) was mixed with 20 mL of water and 20 mL of sunflower oil. The mixture was emulsified with an Ultra-Turrax homogenizer (Model T18 basic, IKA Labortechnik, Staufen, Germany) at 9500 rpm for 1 min and then centrifuged at 3490× *g* for 30 min (Sorvall RC-5B centrifuge, DuPont Company). A second centrifugation was conducted for 5 min at 3000× *g* (Eppendorf 5810R centrifuge, Eppendorf AG, Hamburg, Germany). The volume of the emulsified layer was measured, and the emulsifying capacity (%) was expressed as the volume of the emulsified layer/total volume. For each flour, the measurements of the emulsifying capacity were conducted in triplicate.

### 2.6. Foaming Capacity and Foam Stability of Flours

The methodology for determining the foaming capacity and foam stability of flours was based on the protocols described by Bencini et al. (1986) [21]. One gramme of flour and 50 mL of water were homogenised using an Ultra-Turrax homogenizer (Model T18 basic, IKA Labortechnik) at 13,500 rpm for 2 min. Immediately after, the height of the foam formed was measured to obtain foaming capacity. For the stability of the foam, the solution was allowed to rest for 30 min, and again the height of the remaining foam was measured. Foaming capacity and foaming stability were expressed as the percentage increase in volume due to the foam. For each flour, foaming capacity and foaming stability measurements were conducted in triplicate.

### 2.7. Viscosity Profile during the Thermo-Mechanical Process of Flours

The viscosity profile was determined during the thermo-mechanical processing of the flours using a starch paste cell adapted to a controlled stress rheometer (AR-G2, TA Instruments, Crawley, England) with a Peltier concentric cylinder system to control the temperature. The flour was mixed with water at a concentration of 10% (*w*/*w*, 25 g total weight). Viscosity values were registered while shearing the mixture at constant shear (160 rpm) and applying the following temperature programme: held at 50 °C for 1 min, heated to 90 °C over 3 min, held at 90 °C for 5 min, and cooled to 25 °C over 5 min. The values of the pasting temperature (temperature value of the first viscosity increment by at least 25 Pa·s over 20 s), maximum viscosity (Pa·s) during heating to 90 °C, and final viscosity (Pa·s) after cooling to 25 °C were obtained from the curves. Each flour was measured in triplicate. The flour pastes obtained were stored at 4 °C for 24 h to evaluate the viscoelastic properties and freeze-thaw stability.

### 2.8. Viscoelastic Properties of Flour Pastes

The viscoelastic properties of the flour pastes were measured using a controlled stress rheometer (RheoStress 1, Thermo Haake, Karlsruhe, Germany), using a 60 mm parallel plate system with a 1 mm gap. Stress sweeps were performed at 1 Hz from 0.001 to 10 Pa to determine the linear viscoelastic region of the samples; then, frequency sweeps were run from 10 Hz to 0.01 Hz. The values of the storage modulus (G′), the loss modulus (G″) and the loss tangent (tan δ) were recorded as a function of frequency. The tests were performed in triplicate.

### 2.9. Syneresis of Freeze-Thaw Stability of Flour Pastes

Freeze-thaw stability of flour pastes from pasting properties analysis was determined using the methodology previously described by Wu et al. (2020) with modifications [22]. The syneresis of the freeze-thaw stability was determined by weighting the flour pastes and subjecting them to a freeze-thaw cycle (freezing at −20 °C for 24 h and thawing in a bath at 30 °C for 2 h). After this, the samples were centrifuged at 528× *g* for 15 min (Eppendorf 5810R centrifuge, Eppendorf AG), and the supernatant was weighed. The percentage of syneresis after freeze-thawing (%) was calculated as g of supernatant/g of flour paste. For each flour paste, a syneresis of freeze-thaw stability measurements was conducted in triplicate.

### 2.10. Instrumental Texture Properties of Flour Gels

Gels were prepared by mixing flour and water (15% w/w, 500 g total weight) with a Heidolph stirrer (Type RZR 1, Schwabach, Germany) at 130 rpm while heating for 25 min in a water bath at 90 °C. After heating, the amount of evaporated water was added to the mixture while gently stirring for another 5 min at 60 rpm and decreasing the temperature of the sample to 50 °C. Finally, the sample was poured into two glass containers (height = 40 mm; diameter = 70 mm), covered with plastic film, and stored at 4 °C for 24 h. Two batches were prepared per sample. The gel cylinders were cut into cylinder-shaped probes: height = 17 mm; diameter = 17 mm.

Instrumental texture measurements were performed at 20 °C with a TA-XT2 plus Texture Analyser (Stable Micro Systems Ltd., Godalming, UK). For each test, four cylinders from two batches were analysed.

#### 2.10.1. Compression up to the Rupture Test

The gel cylinder was compressed at a speed of 1 mm/s up to 50% of its original height to ensure rupture with a 35 mm diameter cylindrical aluminium plunger. The Young’s modulus was calculated as the result of the division between the true rupture stress (σ*_F_*, N/mm^2^) and the true rupture strain (ε*_F_*, dimensionless) (Equation (1)):

(1) $$E=\frac{\sigma_{F}}{\varepsilon_{F}}$$

The true rupture stress (σ*_F_*) and the true rupture strain (ε*_F_*) were calculated using Equations (2) and (3) [23]:

(2) $$\sigma_{F}=F\times \left(\frac{h_{0}-∆h}{A_{0}h_{0}}\right)$$

(3) $$\varepsilon_{F}=\ln\left(\frac{h_{0}}{h_{0}-Ah}\right)$$

where *F* is the maximum rupture force and ∆*h* is the height variation of the sample height during compression (both obtained from the force-time curve); *h*_0_ is the initial height area of the sample, and *A*_0_ is the cross-sectional area of the sample.

#### 2.10.2. Texture Profile Analysis (TPA)

The gel cylinder was compressed twice, up to 10% of its original height, at a speed of 1 mm/s with a 35 mm diameter cylindrical aluminium plunger. From the force-time curves [24], the values of hardness (maximum force during the first compression cycle, N) and springiness (the height that the food recovers during the time between the end of the first bite and the start of the second bite, dimensionless) were obtained.

### 2.11. Data Analysis

A one-way analysis of variance (ANOVA) was used to study the effect of the type of flour on each technological and rheological parameter. The significance of the differences between the mean values was determined by post-hoc Fisher tests (α = 0.05).

To compare flours, principal component analysis (PCA) with varimax rotation was applied to the correlation matrix of the techno-functional properties of flours (swelling capacity, water holding capacity, oil holding capacity, emulsifying capacity, foaming capacity, and foaming stability) and separately to the correlation matrix of the rheological properties of flour pastes (pasting temperature, maximum viscosity during heating (90 °C), final viscosity after cooling (25 °C); G′, G″, and tan δ, and finally syneresis after freeze-thawing). The proximate composition data was included in the supplementary variables table. All calculations were performed with XLSTAT version 2020.4.1 software (Addinsoft, Paris, France).

## 3. Results

### 3.1. Proximate Composition of Flours from Different Vegetable Sources

The proximate composition of the seven flours (chickpea, lentil, red lentil, white bean, quinoa, amaranth, and oat) is shown in Table 2. The protein content of the flours, ranging between 11.6% and 25.9%, varied significantly (*p* < 0.01) depending on the source of origin. For red lentil and lentil flour, the protein content was significantly higher than for the rest of the flours, and for oat flour, the protein content was lower than for the rest of the flours. Although the percentage of fat was low for all the studied flours (from 1.4% to 6.3%), it varied significantly depending on the source (*p* < 0.01). The fat content was significantly higher for chickpea flour and significantly lower for lentil flour than from other sources. Dietary fibre content varied greatly among flours depending on the source (*p* < 0.01). The fibre content was much higher (more than twice as high) for white bean flour (33.5%) than for the other six flours. It was intermediate for lentil and oat flour (16.4% and 15.3%) and low for quinoa and red lentil flour (8.7% and 9.8%, respectively). The carbohydrate content ranged from 31.3% to 57.4% and significantly depended on the type of flour (*p* < 0.01). Quinoa, oats, and amaranth were the three flours with a higher carbohydrate content (>55%), whereas white bean flour had only 31.3% of carbohydrates.

The pulse flours had the highest protein content compared to other flours. The fat content was low for the vegetal flours, with chickpea flour being the highest. The white bean flour had the highest amount of fibre compared to the other flours, which had less than half of the amount in white bean flour. Quinoa, oat, and amaranth flours had the highest content of carbohydrates compared with the other pulse flours.

### 3.2. Techno-Functional Properties of Flours from Different Vegetable Sources

The mean values of the techno-functional properties of the flours (swelling, holding capacity of water and oil, emulsifying and foaming capacity, and foaming stability) are shown in Table 3.

Swelling capacity values, corresponding to the volume occupied by fully hydrated flour, ranged between 2.49 and 3.99 mL/g and varied significantly with the type of flour (*p* < 0.01). White bean flour had higher swelling capacity values than the rest of the samples. Chickpea and red lentil were the flours with the lowest swelling capacity values.

Water holding capacity values, corresponding to the grammes of water held per gramme of flour, ranged from 0.72 to 1.64 g/g and varied significantly with the type of flour (*p* < 0.01). White bean and oat flours retained more water, and the red lentil flour retained less water than the rest of the flours.

Oil holding capacity, which corresponds to the grammes of oil held per gramme of flour, ranged from 0.69 to 0.97 g/g and significantly varied with the type of flour (*p* < 0.01). The oat and quinoa flours had higher oil-holding values than the rest of the flours.

The emulsifying capacity values (volume of the emulsion layer) varied highly (0.5–51.4%) depending on the type of flour (*p* < 0.01). Pulse flours (chickpea, lentil, red lentil, and white bean) showed a high emulsifying capacity, as they formed an emulsion layer that occupied 50% of the volume of the water and oil mixture. In contrast, the quinoa, amaranth, and oat flours formed only a thin emulsion layer that occupied less than 14% of the volume.

The foaming capacity (volume of the foam layer) and foaming stability (volume of the foam layer remaining after 30 min) varied greatly depending on the type of flour (*p* < 0.01). The volume of foam that formed was very high for red lentil and lentil flours (>55%), high for chickpea and white bean (46–49%), and intermediate for quinoa (34%). For these flours, the foam was stable, as 70% of its volume remained after 30 min, except for the white bean, where more than half of the initial foam disappeared after 30 min. The red lentil and lentil flour produced the highest amount of foam that was also stable. However, amaranth and oat flour produced little foam.

**Figure 1:**
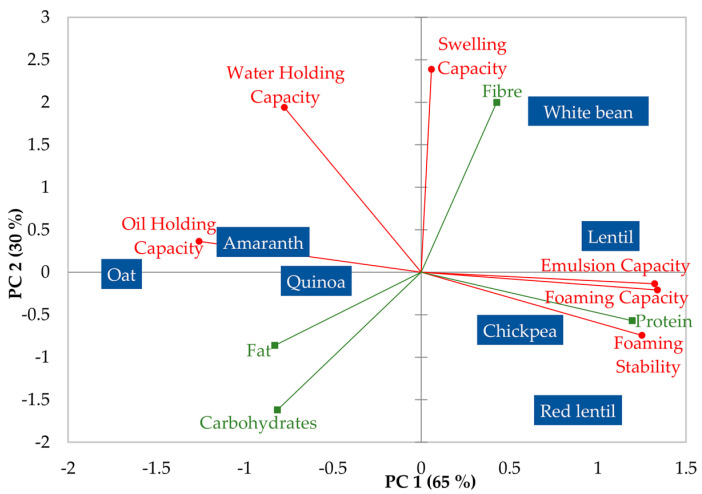
PCA plot showing variation in flours from different vegetable sources according to their techno-functional properties. The proximate composition variables were included as supplementary variables.

PCA was used to summarise the variation in these functional properties among the seven vegetal flours. The PCA plot of the two first components that account for 95% of the total variability is shown in Figure 1.

The first component (65% of variability) explained differences in foaming, emulsion, and oil holding capacity of flours. On the right, chickpea, lentil, red lentil, and white bean flours are differentiated for their high emulsifying and foaming capacity against their low oil holding capacity. On the left, the oat, amaranth, and quinoa flours had high oil holding capacity but low emulsifying and foaming capacity. Composition variables were also included in the PCA as supplementary variables to explain differences in the functionality of flours. The protein content appeared related to the first factor, explaining the differences in emulsifying, foaming, and oil holding capacity among flours. Proteins are good at emulsifying and foaming because they lower surface tension at the interface (air/water or oil/water) and form a layer that maintains separated air bubbles or oil droplets and avoids coalescence [25]. Increasing protein concentration increases the rate of protein adsorption at the interfacial layer and makes it thicker [26,27]. A cohesive, elastic, and viscous interfacial layer favours foam and emulsion formation and stability [28]. Therefore, emulsifying and foaming ability are high for protein-rich pulse flours, whereas oat and amaranth flours cannot emulsify or foam due to their low protein content (<15%), which is probably below the critical concentration needed to surround the droplet or bubble surface. However, it should be considered that interfacial properties are also determined by the nature of the protein (surface hydrophobicity or solubility), and the observed differences could also be attributed to the different types of protein. According to a previous study, oat globulins contain glutamine-rich regions at the surface that make them more hydrophobic compared to other globulins [6], and then at natural pH, oat proteins have lower solubility and poorer interfacial properties compared to soy and pea [29]. Amaranth protein isolates also showed poor emulsifying and foaming capacity compared to soy protein due to a lower solubility at neutral pH [30,31]. The higher oil holding capacity appears also to be related to the more hydrophobic character of proteins in oat, amaranth, and quinoa flours [32], as oil retention is primarily attributed to the physical entrapment of oil that binds to nonpolar side chains in the amino acids of proteins.

The second component that explains swelling and water holding capacity mainly separates pulse flours: white bean flour (on top) with the highest values of these parameters and chickpea and red lentil (on the bottom) with the lowest swelling and water holding capacity. The variable composition related to this second component is the fibre content. Fibre content explained the large differences in swelling and water holding capacity observed between the white bean and red lentil flours. As observed for other flours, fibre can greatly absorb water due to the abundance of hydroxyl groups in polysaccharides, which allows for more water interaction through hydrogen bonds [33]. However, the fibre content did not explain other variations observed among the rest of the flours, which may be attributed to factors like protein, starch, and particle size that can be different among flours and are also related to water adsorption and retention [10,11].

### 3.3. Pasting Behaviour of Flours and Rheological Properties of Pastes and Gels

**Figure 2:**
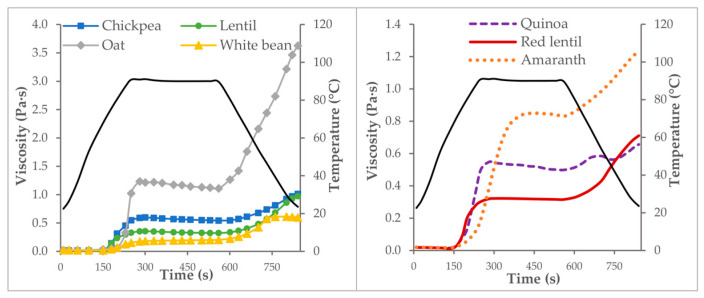
Viscosity curves of flour/water mixtures (10%) as a function of temperature (black line) and time.

Figure 2 shows the changes in the viscosity of the flour dispersions (10%) during the heating process. At the beginning, the viscosity values of the mixture remained constant until a certain temperature (the pasting temperature) was reached, where the viscosity suddenly and sharply increased to reach viscosity values that remained almost constant during the heating period at 90 °C. During cooling, the viscosity progressively increased until it reached the maximum viscosity values at 25 °C, which corresponded to the viscosity of the paste formed. However, the holding period (90 °C) in the pasting test is commonly associated with a decrease in viscosity because of the breakdown of swollen starch granules at high temperatures and mechanical shear [22,34,35]. Nevertheless, none of the flours in this study showed an important decrease in viscosity values during heating, indicating the ability of the starches to withstand high temperatures and shearing.

The pasting profiles were different among the vegetable flours, and, for comparison, three parameters were obtained: pasting temperature, maximum viscosity during heating, and final viscosity. The mean values for these parameters are shown in Table 4. The pasting temperature ranged from 72.8 to 82.9 °C and varied significantly with the type of flour (*p* < 0.01). Quinoa, chickpeas, and lentils had a lower pasting temperature, so they gelatinised sooner, whereas amaranth and oats needed a higher temperature to start the gelatinisation process. Maximum viscosity during heating (90 °C) varied greatly (0.21 and 1.21 Pa·s) with the type of flour (*p* < 0.01). The dispersions of amaranth and oat flour reached viscosity values much higher (0.84 and 1.21 Pa·s) than the rest of the flours and were three times the viscosity values of white bean, red lentil, and lentil (0.21–0.36 Pa·s).

After cooling, viscosity values at 25 °C ranged from 0.59 to 3.58 Pa·s and varied significantly depending on the type of flour (*p* < 0.01). The oat flour paste showed again the highest viscosity value, which was over three times higher than the other flours. White bean and quinoa flours produced pastes with lower viscosity at the end of the elaboration process.

**Figure 3:**
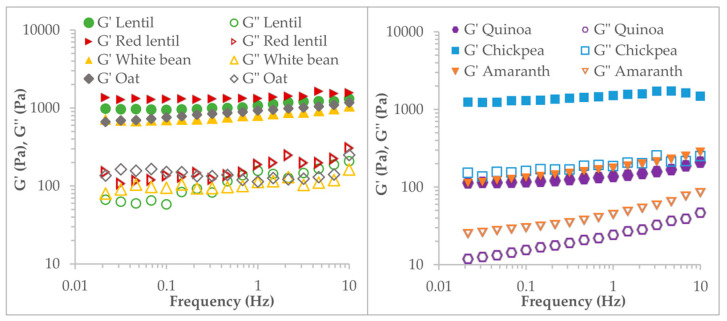
Mechanical spectra of flour pastes (10%). G′ (filled symbols) and G″ (open symbols).

After 24 h, the viscoelastic properties of the flour pastes were also studied. In Figure 3, mechanical spectra reflect the viscoelastic properties of the flour pastes. All showed typical mechanical spectra of gel structures with storage modulus (G′) values over loss modulus (G″), but the values for both moduli were different between flours (Table 5).

The values of G′, G″, and loss tangent (tan δ) were taken at a frequency of 1 Hz to compare flours. The three parameters were shown to vary significantly with the type of flour (*p* < 0.01). G′ and G″ were higher for chickpea, lentil, and red lentil flour pastes than for quinoa and amaranth, with the lowest values. The loss tangent was much higher for the amaranth flour paste (0.32), indicating a system with a higher relative contribution of viscous behaviour than for the rest. Along with the low values of G′ and G″, the amaranth flour pastes form a weak gel system. However, the red lentil and chickpea flour pastes showed low tan δ values (0.12–0.13), indicating a more structured system with a greater contribution to solid behaviour.

**Figure 4:**
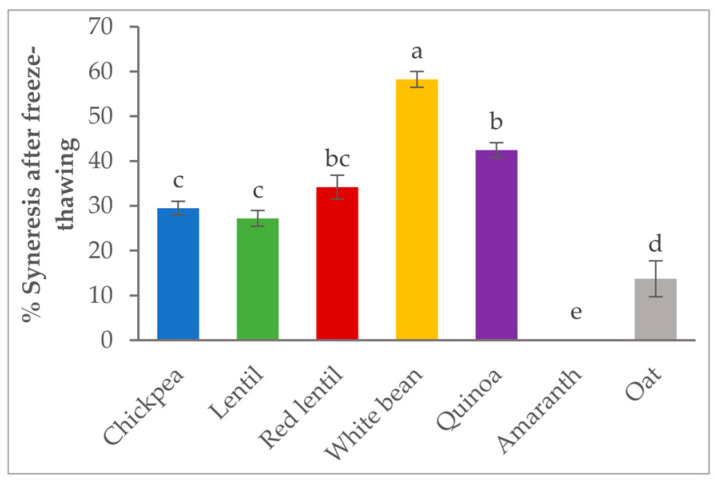
Percentages of syneresis after freeze-thawing of flour pastes. Values not sharing letters are significantly different according to Fisher’s LSD test (*p* < 0.05).

In Figure 4, the stability of flour pastes after a freeze-thaw process was studied by measuring syneresis (the amount of water lost). Good freeze-thaw stability corresponds to a low percentage of syneresis [36]. The amount of lost water varied greatly depending on the type of flour (*p* < 0.01). It was high for white bean and quinoa flour pastes (58% and 43%, respectively), indicating poor stability. However, the syneresis was very low for amaranth and oat, indicating that they were the more stable flour pastes.

White bean, quinoa, amaranth, and oat flours could not form a self-supporting gel in the range of concentrations assayed (15–30%). Chickpea, lentil, and red lentil flours formed self-supported gels at a concentration of 15%. The texture of these gels was characterised by three parameters: Young’s modulus, hardness, and springiness (Table 6). For the three parameters, the values varied significantly with the type of flour (*p* < 0.01), being higher for chickpea and lentil than for red lentil. According to these results, chickpea gels are stiffer, harder, and more elastic, while red lentil gels are malleable, softer, and more pliable.

**Figure 5:**
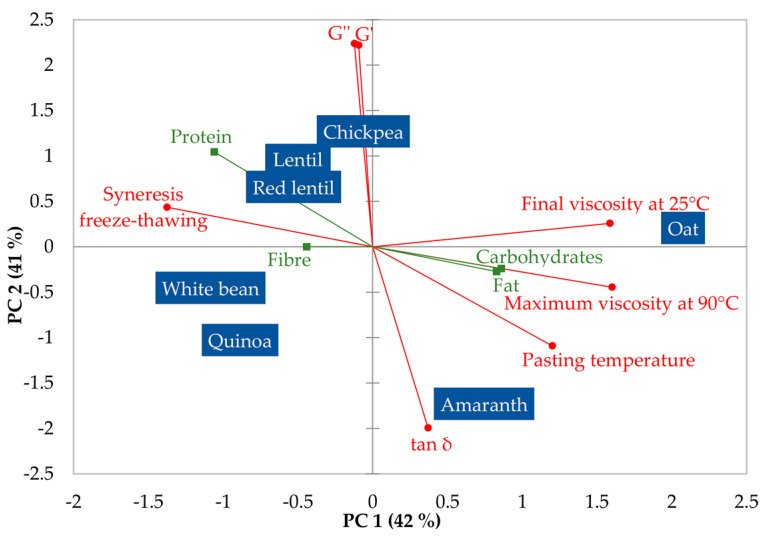
PCA plot showing the variation in the thermal properties of flours from different vegetable sources. Proximate composition variables were included as supplementary variables.

In Figure 5, the PCA plot summarises the variation in the pasting properties of the flours and the rheological properties of the flour pastes. The first component (42% of variability) explains differences in the viscosity of flour pastes (during preparation) and syneresis values (after freeze-thawing). On the right side, oat and amaranth flours are differentiated because they produce high-viscosity pastes that are stable to the freeze-thaw process, whereas the white bean flour paste (on the left) had low viscosity and stability values. Chickpea, lentil, red lentil, and quinoa flours showed intermediate values for these properties. The second component explains differences in the viscoelastic parameters of flour pastes and separates (on top) chickpea, lentil, and red lentil with higher values of both G′ and G″ moduli and low tan δ values that indicate a more elastic structure on these pastes, which coincides with the fact that chickpea, lentil, and red lentil flours were the only ones capable of forming self-standing gels. At the bottom appeared amaranth and quinoa, which form flour pastes with a more fluid-like structure. The variables of proximate composition in the plot showed short vectors except for the protein content, which showed to be correlated mainly to the first component but also to the second component. This indicates that differences in thermal and rheological properties are not well explained by the quantitative differences in composition and are more complex and related to the qualitative differences in composition, such as the type of starch and protein.

The increase in viscosity in flour dispersions during heating is mainly attributed to starch gelatinisation; the starch granules swell because they absorb water molecules. Furthermore, there is a loss of crystalline regions of amylopectin that continue to incorporate water, and eventually amylose is leached into the continuous phase and the structure of the starch granule may disappear [12,37]. The gelatinisation and swelling properties of starch vary depending on vegetable sources due to their differences in the length and branching degree of the amylopectin chain, the amylose to amylopectin ratio, and the granule architecture (crystalline to amorphous ratio) [38]. In addition, other components of flour, such as proteins or lipids, may also affect the gelatinisation process of starch. In our study, the oat flour paste developed high viscosity, which, according to previous research [39], is due to its high starch content but also to the high content of *β*-glucans, which have shown a high capacity to increase viscosity in starch dispersions. In this study, the protein content shows a correlation with the pasting properties of the flours, as those with higher protein contents, such as pulses and quinoa, had lower viscosity values. The decrease in pasting properties of starch regarding protein contents has been observed [40] and has been attributed to competition for water between proteins and starch granules, as proteins can limit the swelling of starch granules during heating.

Differences in the viscoelastic properties of flour pastes and the texture of gels after storage showed a different trend from viscosity, probably due to the increased relevance of the protein gelation phenomenon. During heating, protein molecules unfold above the denaturation temperature and then can aggregate and form a gel that also entraps water [41]. The final structure then comprises remnant granules, the interaction of the leached-starch polymers, and the aggregated protein molecule network. In this study, the elastic behaviour of the flour pastes and the hardness of the gels are partially related to the protein content of the flours, probably because protein gelation occurs above a critical concentration [42] and is more pronounced as the concentration increases. This explains why chickpea, lentil, and red lentil flours produced pastes with higher elastic contributions and self-supporting gels, whereas amaranth and oat flours showed more fluid-like pastes and low gelation abilities. However, white bean and quinoa flours, with a similar protein content as the other pulses, showed pastes with lower elastic contributions and poor gelling ability, indicating that other factors such as the type of protein [43] also determine that gelation may influence the structure of pastes and gels. The white bean and quinoa flours also showed poor freeze-thaw stability; this may be due to their low starch gelatinisation and low protein gelation, as well as the formed structure’s lower ability to retain water molecules.

## 4. Conclusions

The techno-functional properties of vegetable flours, pastes, and gels highly varied among the seven sources studied (chickpea, lentil, red lentil, white bean, quinoa, amaranth, and oat). The differences were partly explained by the proximate composition (protein and fibre content), and some could be attributed to the types of protein and starch that vary between sources.

Pulse flours provide high emulsifying and foaming capacity, which is explained by the high protein content of these flours. Chickpea, lentil, and red lentil produce flour pastes with a more elastic character and form hard, self-standing gels. White bean flour has a high water holding capacity due to its high fibre content and produces more fluid flour pastes with low freeze-thaw stability that do not form gels.

Quinoa, amaranth, and oat flours show a low emulsifying and foaming capacity, explained by the low protein concentration but also by the hydrophobicity and low solubility of the proteins. Oat and amaranth show high viscosity pastes with a low elastic character and are very stable to freeze-thawing. However, the quinoa flour showed flour pastes with low viscosity and low elastic contribution. None of the three flours formed a self-standing gel.

This research shows that different sources of vegetal protein offer a wide range of functionalities and gives insight into their suitability as ingredients to develop plant-based products. The mixing of some of these different flours is expected to also expand the opportunities to obtain combined functionalities in the product.

Further studies considering the qualitative analysis of components (starch and protein) from different sources would help to explain the different properties of vegetable flours. It should also be considered that, in this study, the described properties correspond to the natural condition of the flour. Additional studies on how pH and ionic strength conditions may also help to understand differences in functionality of different vegetable flours and also provide a broader range of applications for them.

## Figures and Tables

**Table 1:** Flour particle size distribution (%) and moisture content (%).

Flour Granulation (%) for Each Mesh Size	Chickpea	Lentil	Red Lentil	White Bean	Quinoa	Amaranth	Oat
>354 µ	25.2	7.5	0.5	3.5	0.0	49.8	67.5
125–354 µ	56.1	51.0	46.6	73.1	84.3	44.0	31.2
90–125 µ	9.7	17.5	17.3	8.3	8.1	4.0	0.8
<90 µ	9.0	24.0	35.6	15.1	7.6	2.2	0.5
Moisture content (%)	8.8	7.9	8.8	10.2	10.6	11.3	10.6

**Table 2:** Mean values of the proximate composition of flours. Standard deviation in brackets.

Flours	Protein (%)	Fat (%)	Fibre (%)	Carbohydrates (%)
White bean	19.2 ^bc^ (2.9)	2.1 ^d^ (0.2)	33.5 ^a^ (0.5)	31.3 ^d^ (3.4)
Chickpea	16.9 ^c^ (2.0)	6.3 ^a^ (0.4)	14.0 ^c^ (1.1)	51.1 ^bc^ (1.0)
Lentil	24.0 ^ab^ (2.8)	1.4 ^e^ (0.3)	16.4 ^b^ (0.9)	48.1 ^c^ (3.4)
Red lentil	25.9 ^a^ (1.2)	2.9 ^c^ (0.1)	9.8 ^e^ (0.6)	50.4 ^bc^ (1.2)
Quinoa	16.2 ^cd^ (2.0)	4.6 ^b^ (0.5)	8.7 ^e^ (0.4)	57.4 ^a^ (0.6)
Amaranth	14.9 ^cd^ (0.6)	4.9 ^b^ (0.1)	11.8 ^d^ (1.1)	54.7 ^ab^ (1.4)
Oat	11.6 ^d^ (2.4)	5.1 ^b^ (0.1)	15.3 ^bc^ (0.5)	55.6 ^ab^ (2.4)

Values not sharing letters are significantly different according to Fisher’s LSD test (*p* < 0.05).

**Table 3:** Mean values of the techno-functional properties of flours. Standard deviation in brackets.

Flours	Swelling Capacity (mL/g)	Water Holding Capacity (g/g)	Oil Holding Capacity (g/g)	Emulsifying Capacity (%)	Foaming Capacity (%)	Foaming Stability (%)
Chickpea	2.90 ^bc^ (0.64)	1.00 ^d^ (0.01)	0.77 ^bc^ (0.01)	51.4 ^a^ (0.7)	46.2 ^b^ (5.7)	32.6 ^ab^ (5.4)
Lentil	3.23 ^b^ (0.21)	1.31 ^c^ (0.07)	0.69 ^c^ (0.04)	46.2 ^c^ (3.3)	57.1 ^a^ (3.1)	43.2 ^a^ (10.6)
Red lentil	2.49 ^c^ (0.10)	0.72 ^e^ (0.01)	0.69 ^c^ (0.01)	49.7 ^ab^ (3.3)	57.9 ^a^ (5.1)	42.5 ^a^ (3.9)
White bean	3.99 ^a^ (0.26)	1.64 ^a^ (0.03)	0.78 ^bc^ (0.03)	47.7 ^bc^ (2.1)	48.7 ^ab^ (9.0)	23.0 ^b^ (9.0)
Quinoa	3.09 ^b^ (0.07)	1.43 ^bc^ (0.17)	0.93 ^a^ (0.11)	13.8 ^d^ (1.2)	34.3 ^c^ (10.0)	24.1 ^b^ (3.6)
Amaranth	3.23 ^b^ (0.33)	1.58 ^ab^ (0.06)	0.85 ^ab^ (0.10)	3.3 ^e^ (0.4)	13.6 ^d^ (4.8)	4.9 ^c^ (2.8)
Oat	3.05 ^b^ (0.11)	1.60 ^a^ (0.13)	0.97 ^a^ (0.09)	0.5 ^e^ (0.4)	0.0 ^e^ (0.0)	0.0 ^c^ (0.0)

Values not sharing letters are significantly different according to Fisher’s LSD test (*p* < 0.05).

**Table 4:** Mean values of the pasting temperature, maximum viscosity at 90 °C, and final viscosity at 25 °C of flours from the testing of thermo-mechanical processes. Standard deviation in brackets.

Flours	Pasting Temperature (°C)	Max. Viscosity at 90 °C (Pa·s)	Final Viscosity at 25 °C (Pa·s)
Chickpea	73.3 ^d^ (0.3)	0.59 ^c^ (0.02)	1.01 ^c^ (0.03)
Lentil	73.2 ^d^ (0.2)	0.36 ^e^ (0.01)	0.99 ^c^ (0.02)
Red lentil	75.6 ^c^ (0.1)	0.34 ^e^ (0.02)	0.76 ^d^ (0.04)
White bean	78.9 ^b^ (1.4)	0.21 ^f^ (0.01)	0.59 ^f^ (0.01)
Quinoa	72.8 ^d^ (0.2)	0.55 ^d^ (0.01)	0.66 ^e^ (0.02)
Amaranth	82.7 ^a^ (0.0)	0.84 ^b^ (0.01)	1.23 ^b^ (0.01)
Oat	82.9 ^a^ (0.1)	1.21 ^a^ (0.02)	3.58 ^a^ (0.07)

Values not sharing letters are significantly different according to Fisher’s LSD test (*p* < 0.05).

**Table 5:** Mean values of the storage modulus (G′), loss modulus (G″), and loss tangent (tan δ) values at 1 Hz of the flour pastes. Standard deviation in brackets.

Flours	G′ (Pa)	G″ (Pa)	tan δ (G″/G′)
Chickpea	1609 ^a^ (101)	206 ^a^ (24)	0.13 ^c^ (0.01)
Lentil	1386 ^a^ (82)	187 ^ab^ (12)	0.14 ^bc^ (0.01)
Red lentil	1432 ^a^ (42)	168 ^b^ (17)	0.12 ^c^ (0.03)
White bean	625 ^b^ (40)	98 ^c^ (12)	0.16 ^bc^ (0.02)
Quinoa	122 ^c^ (27)	21 ^d^ (5)	0.17 ^b^ (0.01)
Amaranth	92 ^c^ (6)	29 ^d^ (6)	0.32 ^a^ (0.05)
Oat	775 ^b^ (24)	108 ^c^ (10)	0.14 ^bc^ (0.01)

Values not sharing letters are significantly different according to Fisher’s LSD test (*p* < 0.05).

**Table 6:** Mean values of the Young’s modulus, hardness, and springiness of flour gels. Standard deviation in brackets.

Flours	Young’s Modulus × 10^−2^ (N/mm^2^)	Hardness (N)	Springiness
Chickpea	2.51 ^a^ (0.3)	0.66 ^a^ (0.02)	0.83 ^a^ (0.01)
Lentil	2.30 ^b^ (0.3)	0.64 ^a^ (0.03)	0.81 ^a^ (0.01)
Red lentil	1.27 ^c^ (0.3)	0.38 ^b^ (0.05)	0.66 ^b^ (0.02)

Values not sharing letters are significantly different according to Fisher’s LSD test (*p* < 0.05).

## Data Availability

Data are contained within the article.
